# The Effect of Oxygen Content in Binderless Cokes for High-Density Carbon Blocks from Coal Tar Pitch

**DOI:** 10.3390/ma14081832

**Published:** 2021-04-07

**Authors:** Seungjoo Park, Seon Ho Lee, Song Mi Lee, Jin-Woo Park, Sung-Soo Kim, Doo-Hwan Jung

**Affiliations:** 1 Fuel Cell Laboratory, Korea Institute of Energy Research (KIER), Daejeon 34129, Korea; tmdwn0903@kier.re.kr (S.P.); pirsys@kier.re.kr (S.H.L.); songmi@kier.re.kr (S.M.L.); parkjw@kier.re.kr (J.-W.P.); 2 Department of Energy Science and Technology, Graduate School of Energy Science and Technology, Chungnam National University, Daejeon 34134, Korea; 3 Department of Chemical and Biomolecular Engineering, Yonsei University, Seoul 03722, Korea; 4 Advanced Energy and System Engineering, University of Science and Technology (UST), Daejeon 34113, Korea

**Keywords:** binderless coke, oxygen up-take, beta-resin, carbonization, swelling

## Abstract

High-density carbon blocks are much lighter than metals and have excellent mechanical properties and are one of the materials garnering attention to replace existing metal parts. In this study, a binderless coke was produced by changing the flow rates of nitrogen and air as a carrier gas during heat treatment of coal tar pitch and using this, a green body was formed at 150 MPa and carbonized to produce a high-density carbon block. We express the binderless coke produced in this way by N10A0, N7A3, N5A5, N3A7, N0A10 according to the ratio of nitrogen and air, and in the case of carbon block, we have added CB in front of it. We then considered the effect of oxygen content in the binderless cokes on the optical, chemical, and mechanical properties. It was observed that the produced binderless cokes develop into a dense mosaic structure with a small particle size as the air flow rate increased. To survey the change in oxygen content of the produced binderless coke, O1s and C1s regions were measured using X-ray photoelectric spectroscopy (XPS), and O1s/C1s was calculated. The O1s/C1s ratio steadily increased as the air flow rate increased, and in the case of N0A10, it increased about twice as much as that of N10A0 to 11.20%. β-resin has a very large effect on the mechanical strength of the carbon block in addition to air in the pitch. And in the case of CB-N0A10, it shows the best mechanical strength with a density of 1.72 g/cm^3^, bending strength of 87 MPa, and shore hardness of 93 HSD.

## 1. Introduction

High-density carbon blocks are much lighter than metals and have excellent mechanical properties, and accordingly are receiving attention as materials that can replace existing metal parts. At present, environmental regulations have become stricter in light of environmental pollution, and it has become essential to reduce the weight of transportation vehicles. To this end, carbon blocks are used in automobiles, aircraft, rockets, etc., to improve fuel efficiency and are also used in various heat-dissipating materials, heat insulating materials, etc., based on their excellent thermal properties [1,2,3,4].

There are several methods for manufacturing high-density carbon blocks, but depending on the number of raw materials, they can be divided into primary and binary systems. Primary systems undergo self-sintering caused by β-resin and require only one material. Additional binder materials are thus not required. On the other hand, binary systems require two materials, a filler and a binder [5,6,7]. Mesocarbon microbeads (MCMBs), which undergo self-sintering and can be molded without additional binders, are typical examples of a primary system [8,9,10]. This is because they use a binder called β-resin. The solubility of a substance varies depending on the type of solvent [11]. This also applies to carbon materials, such as coke and pitch. Since the carbon material is generally dissolved in an organic solvent. Further, these are not one compound but a mixture of several compounds, but the molecular weights of the mixed compounds are different so that the solubility differs depending on the solvent. β-resin can be defined from these properties. Generally, β-resin is defined as substances that are dissolved in quinoline but not in toluene [12], but there are different definitions depending on the researcher.

β-resin has a fluid phase, and it can play the role of firmly attaching the solid phase by entering the empty spaces in the primary system. In addition, volume shrinkage is caused during sintering, and high density can be achieved [13,14,15]. On the other hand, binary systems that do not have β-resin include needle coke and graphite, which require binders during molding. To improve the mechanical properties after heat treatment, impregnation is performed. In addition, carbon nanotubes (CNTs), carbon fiber, carbon black, etc., can be added to impart specific physical properties [16,17]. A high-density carbon block is manufactured through a carbonization process in which the green body thus formed is heat-treated at 800 to 1500 °C, and a graphitization process in which the carbonized body is heat-treated at 2000 °C or higher [18].

To ensure high mechanical properties of the carbon block, swelling suppression technology is required. Swelling is a phenomenon that occurs mainly in the process of carbonization, where the volatile matter remaining in the carbon block is rapidly released to the outside, and this causes swelling and pore formation [19]. Due to this phenomenon, the density decreases, the porosity increases, and the mechanical properties deteriorate. As a result, many studies have been conducted to prevent this. Mochida et al. oxidatively stabilized the raw material before molding to obtain a heat treatment effect and removing volatile matter. Consequently, it is reported that the test piece does not deform even at high temperatures, and swelling can be suppressed [20]. In addition, Ragan et al. added hydroxy groups, carbonyl groups, etc., which contribute to sintering by oxidizing needle-coke and then mixed this with coal tar binder pitch for molding and heat treatment. Further, it has been reported that when molded into needle coke, which has the largest amount of up-taken oxygen compared to the amount of escaped oxygen, depending on the degree of oxidation of needle coke, it has the highest mechanical properties [14]. In this study, we considered the effect of the oxygen content of binderless coke produced from coal tar pitch on the mechanical properties of high-density carbon blocks. Nitrogen was used as a carrier gas by changing the flow rate of air, while producing a binderless coke that does not require a binder pitch or impregnation process. The chemical and mechanical properties and microstructure of the green body produced from this and a heat-treated carbon block were investigated. The microstructure was measured using scannig electron microscopy (SEM), and the chemical properties were measured by examining changes in the content of fixed carbon and volatile matter via a proximate analysis (PA) and thermogravimetric analysis (TGA). The mechanical strength at which the morphology of oxygen up-taken on the surface of the binderless coke was grasped using X-ray photoelectric spectroscopy (XPS) and was analyzed using a universal testing machine (UTM) and a shore hardness tester.

## 2. Experimental

### 2.1. Materials

The raw material used a special pitch (JFE Chemical, Tokyo, Japan) with a softening point of 250 °C, and the physical properties are listed in Table 1.

### 2.2. Preparation of High-Density Carbon Blocks

#### 2.2.1. Preparation of Binderless Coke

First, 50 g of raw material flowed through a 60.5Φ × 275 Pyrex tube with 0 to 100 cc/min of air and 0 to 100 cc/min of nitrogen to a total of 100 cc/min, and the temperature was raised by 2 °C/min to 470 °C for 2 h. As a result of conducting a preliminary experiment, high yield and mechanical properties were exhibited when heat-treated at 470 °C. for 2 h, thus this temperature and time were adopted. A binderless coke was produced by heat treatment and was pulverized using a planetary mill and then passed through a 75 μm mesh to produce the binderless coke. The binderless coke produced in this way was denoted as N10A0, N7A3, N5A5, N3A7, and N0A10 according to the flow rate ratio of nitrogen and air.

#### 2.2.2. Green Body Manufacturing and Carbonization

A green body was formed from room temperature at a pressure of 150 MPa using a 35 × 35 × 40 compression mold and heat-treated at 1200 °C for 1 h at a heating rate of 5 °C/min with a horizontal tube furnace and then sintered.

### 2.3. Analysis

The thermal behavior and fixed carbon content of each binderless coke specimen were measured using thermogravimetric analysis (TGA, STA409PC, Netzsch Corp, Selb, Germany) at a heating rate of 5 °C/min to 900 °C in a nitrogen atmosphere. A proximate analysis (PA) was performed to measure changes in volatile matter and fixed carbon content, which was measured with reference to the international standard KS E ISO 1171. The contents of moisture, volatile matter, ash, and fixed carbon were measured, and the contents of moisture were measured after drying at 107 °C for 1 h, and then the weight loss was measured. Volatile content was measured by carbonization at 925 °C for 7 min, and ash was measured by weighing the sample remaining after combustion at 800 ℃. For fixed carbon, the total amount of water, ash, and volatile matter was excluded from 100%. The content of β-resin was checked by ASTM D2318-15 [21] and ASTM D4072-98 [22]. First, 1.000 g of binderless coke was dissolved in 25 mL of quinolone and stirred at 80 °C for 20 min, followed by filtration with a 5 MPa vacuum pump using a 1.6 micrometer filter to measure Quinoline Insoluble (*QI*), and using the same method, Toluene Insoluble (*TI*) was measured.

$$\beta -resin\left(\%\right)\;=\;\frac{TI-QI}{\left(weight\;of\;sample\right)}\times 100$$

The morphology of oxygen functional groups was analyzed using XPS (K-alpha +, Thermo Scientific, Waltham, MA, USA). It can be expected that by flowing air, oxygen gives functional groups, such as hydroxy group (–OH), carbonyl group (–C=O), and carboxyl group (–COOH), access to the edge part of binderless coke, which has relatively high surface energy.

### 2.4. Mechanical Properties

Flexural strength was measured using a Universal Testing Machine (UTM, WL2100, WITHILAB ltd, Daegu, Korea) with reference to ASTM D790-17 [23], and the formula for calculating the flexural strength was as follows:

$$\sigma_{f}=\frac{3\times P\times L}{2\times w\times t^{2}}$$

where *P* is the breaking pressure of the test piece, *L* is the distance between the flexural strength measuring instrument (20 mm), *w* is the width of the specimen (10 mm), and *t* is the thickness of the specimen (3 mm).

Shore hardness was measured using an ASTM D2240 [24] using a shore hardness tester (SH, Type-D, Kobunshi Keiki, Kyoto, Japan). The load was measured by adding 50 N.

The microstructure were observed using an SEM (S-3500N, Hitachi, Tokyo, Japan) observation was performed to observe the denseness of the tissue and how the structure has changed after carbonization at a magnification of 1000 times using an electron beam of 10 kV.

## 3. Results and Discussion

### 3.1. Chemical Properties of Binderless Cokes According to Air Flow Ratio

Table 2 shows the change in the yield of binderless coke according to the flow rate of air. As summarized in Table 2, the yields of binderless cokes decreased when the air flow rate was increased, and similar yields were maintained when the air flow rate was over 30 cc/min. This means that the oxygen in the air reacted sufficiently with C, the main component of the pitch, and as result there were no volatile materials in the cokes at the heat temperature of 470 °C.

This phenomenon occurred in the pitch when the amount of unreacted low molecular weight in the coke was slightly present, and the yield was relatively high when the heat treatment was performed by flowing only nitrogen. Because the low molecular weight substances are thermally stable due to the oxygen cross-linking reaction, such as hydroxyl group, carbonyl group, and carboxyl group, the change in yield was not greatly displayed. A study by Ragan et al. [14] showed similar results.

Table 3 shows the proximate analysis results of the binderless coke produced while changing the air flow rate ratio. The volatile content decreased as the air flow rate increased, and when the air content was 100% (N0A10), it decreased from 8.61 wt% to 6.58% gradually. Conversely, the fixed carbon content gradually increased from 89.44 wt% to 91.46 wt%. It was judged that oxygen in the air, as shown in Table 2, caused a cross-linking reaction to generate hydroxyl, carbonyl, and carboxyl groups [25], and the low molecular weight volatile matter was reduced, and the carbon content was increased. This is known as a typical characteristic of cokes produced through air blowing [26,27,28].

**Figure 1:**
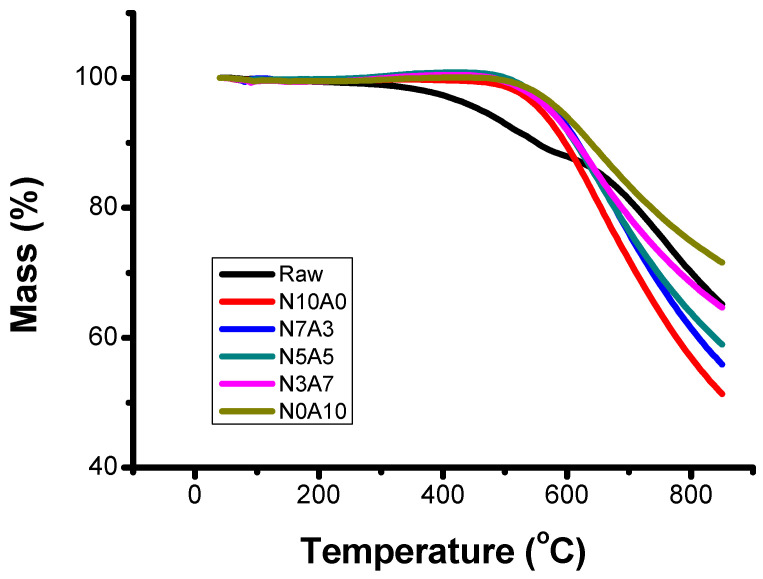
Thermogravimetric analysis (TGA) of binderless cokes manufactured according to flow ratio of nitrogen and air.

Figure 1 shows the TGA attribute value according to the air flow rate ratio. It can be seen that the raw material had a softening point of 250 °C, and as the air flow rate ratio increased, the thermal stability was displayed in the order of N0A10 > N3A7 > N5A5 > N7A3 > N10A0. This means that it had a higher molecular weight and had changed to a thermally stable substance [29,30], and N0A10, N5A5, and N7A3 did not have a large difference starting from a heat-treating temperature of 500 °C, as shown in Table 2. Because the oxygen cross-linking reaction was sufficiently occurring at N7A3, N5A5, N3A7, and N0A10.

**Figure 2:**
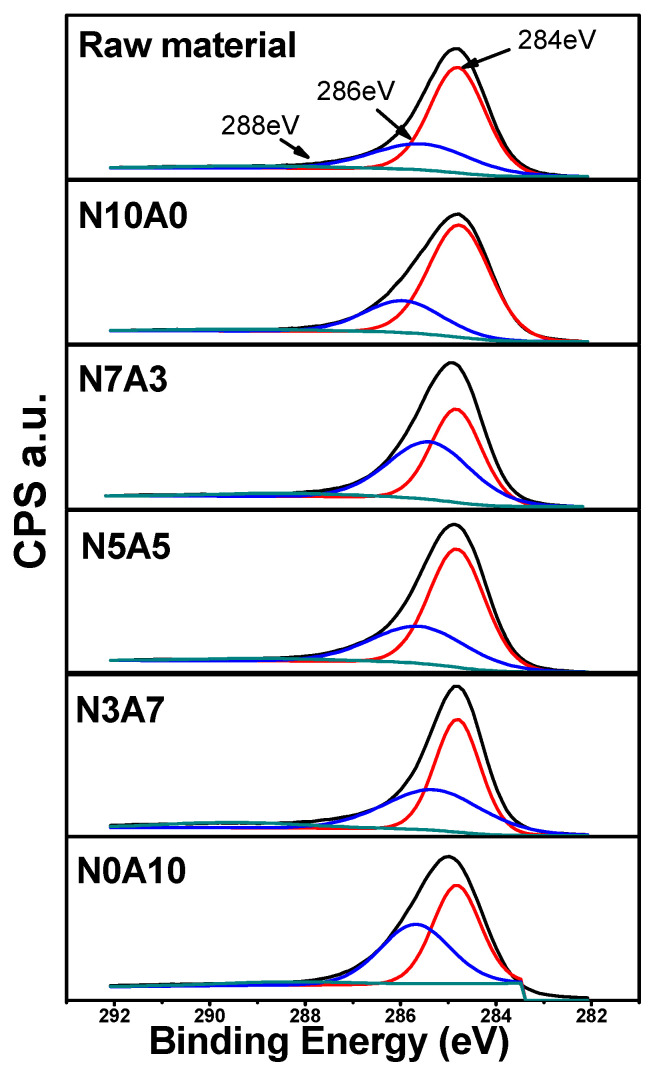
X-ray photoelectric spectroscopy (XPS) C1s of binderless cokes manufactured according to flow ratio of nitrogen and air.

Figure 2 shows a C1s XPS graph of a binderless coke manufactured based on the air flow rate ratio. The XPS graph can be fitted with three peaks, 284 eV indicated C–C, 286 eV indicated C–O, and 288 eV indicated O–C=O. C–O (286 eV) peak and O–C=O (288 eV) peak were sharp and displayed strong intensity as the air flow rate increased. Thus, the content of C–O group and O–C=O group increased [31,32,33]. On the other hand, in the case of N0A10, it was found that the two groups in which the intensity of both peaks appeared weakly decreased, and the amount of oxygen uptake in the form of a functional group. From this phenomenon, the amount of oxygen uptake in the form of a functional group decreased, and while the difference in the amount of oxygen flowing out in the form of CO or CO_2_ decreased up to N5A5. Whereas, when the flow rate of air increased, the amount of C reacting with O and exiting in the form of CO or CO_2_ increased.

**Figure 3:**
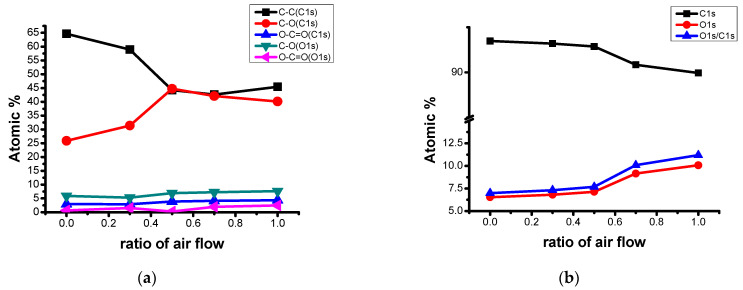
(**a**) Atomic% of the functional group relate to C and O, (**b**) Ratio of O1s/C1s.

Table 4 summarizes the changes in the atomic% of oxygen functional groups according to the air flow rate ratio, and Figure 3a illustrates this graphically. As the air flow rate ratio increased, the C1s atomic% decreased and O1s atomic% increased. In particular, the ratio of O1s/C1s [34,35] showed that the raw material was 6.19%, while N0A10 was about twice as high, as shown in 11.20%. In particular, the atomic% of C–O (C1s) appeared remarkable compared to O–C=O. This was the result of the reaction of oxygen and carbon to produce more hydroxyl groups than the carbonyl group.

**Figure 4:**
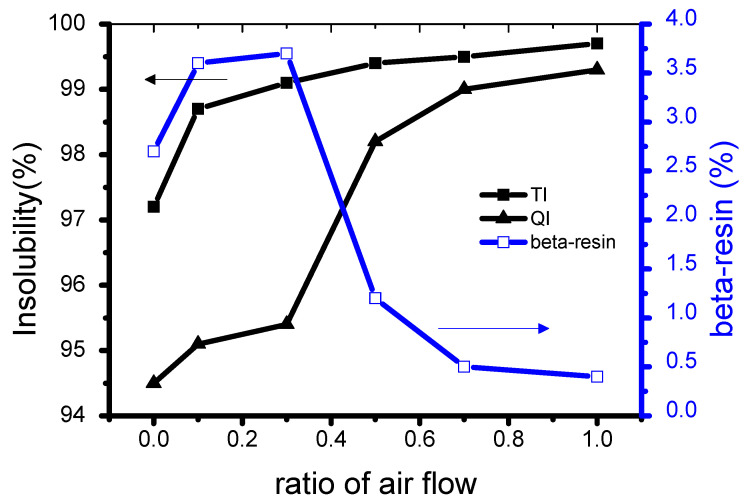
β-resin change in binderless cokes manufactured according to flow ratio of nitrogen and air.

Figure 4 shows the β-resin change in the binderless coke manufactured according to the air flow rate. The heavier the air flow rate was, the more β-resin increased and dereasesd after 30cc/min. This is because the content of TI, which is a relatively low molecular weight substance that is generally known to have a relatively higher reactivity than that of a high molecular weight material, increased with an increase in the flow rate of air. The content of QI, which is a relatively high molecular weight substance, increased relatively slowly compared to TI. Within the scope of this experiment, the highest β-resin value was 3.7% for N7A3.

### 3.2. Mechanical Properties

Table 5 shows the mechanical properties, such as green density, density, flexural strength, and shore hardness, according to the flow rate ratio. The density of green body manufactured while changing the air flow ratio at the same pressure was the highest at 1.365 g/cm^3^ for CB-N0A10 and the lowest at 1.324 g/cm^3^ for CB-N10A0. Such characteristics were obtained because oxygen strengthens the intermolecular binding force and induces it to have a higher density as the flow rate ratio of air increases. Ragan et al. [14] reported similar research results using needle coke and binder pitch.

Characteristics of density after carbonization showed a similar tendency to the green density; CB-N0A10 had the highest value at 1.724 g/cm^3^, and CB-N10A0 had the lowest value at 1.518 g/cm^3^. This means that the physical characteristics of the green body were the main factors that determined the physical characteristics of the high-density carbon block.

Similar to the density characteristics, the flexural strength was the highest at 87 MPa for CB-N0A10 and the lowest at 44 MPa for CB-N10A0, showing the same tendency as the density characteristics.

Shore hardness also showed a tendency similar to other mechanical properties, with CB-N0A10 having the highest shore hardness at 93 and CB-N10A0 having the lowest shore hardness at 62 HSD.

**Figure 5:**
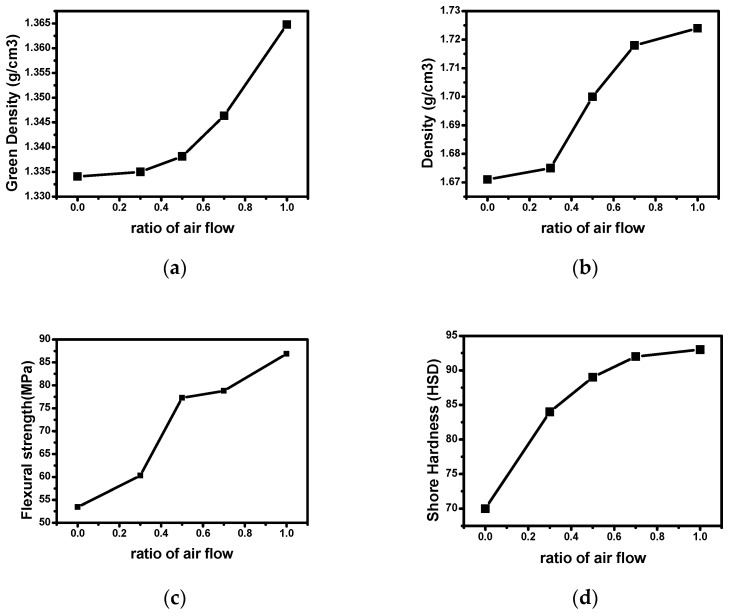
Mechanical properties of carbon blocks according to ratio of air flow (**a**) green density, (**b**) density after carbonization, (**c**) flexural strength, (**d**) shore hardness.

These tendencies are more obviously shown in Figure 5. The more the ratio of air flow, the higher mechanical properties, such as density, flexural strength, and shore hardness.

These mechanical strength properties can be explained via the ratio of O1s/C1s, as shown in Figure 3b. The change in atomic% of C1s and atomic% of O1s and the rate of change can be clearly known, but it can be observed that the O1s/C1s ratio [35] steadily increased as the air flow rate increased. From this, it is judged that the O-related functional groups sufficiently play a role in inducing strengthening of the intermolecular binding force and induce higher mechanical properties [36].

### 3.3. SEM

**Figure 6:**
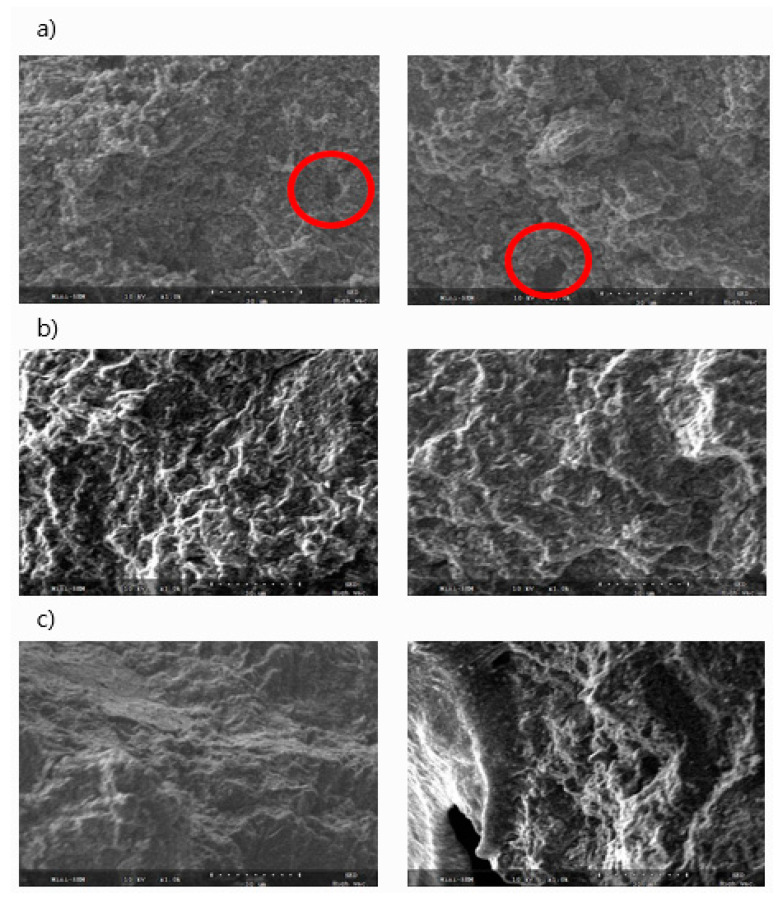
SEM image of high-density carbon blocks (**a**) CB-N10A0, (**b**) CB-N7A3, (**c**) CB-N0A10.

Figure 6 shows these fracture surfaces observed by SEM. In the case of a carbon block in which air was not injected, it can be observed that pores were generated after carbonization. This is because, as shown in Table 2, the content of volatile matter was high, and pores were rapidly formed during the heat treatment process. A large hole can be seen in Figure 5c. It seems that the shape of the hole did not occur due to swelling but the shape of species that was sampled by breaking the test piece. On the other hand, in the case of CB-N7A3 and CB-N0A10, swelling did not occur, and the structure was very dense, but this was realized by adding air, and the oxygen in the air not only imparts functional groups advantageous for sintering but also induces a cross-linking reaction because the volatile matter is removed [14]. In particular, the carbon block manufactured under the conditions of N0A10 showed the best mechanical properties, and it can be seen from the SEM photograph that the structure was the finest.

**Figure 7:**
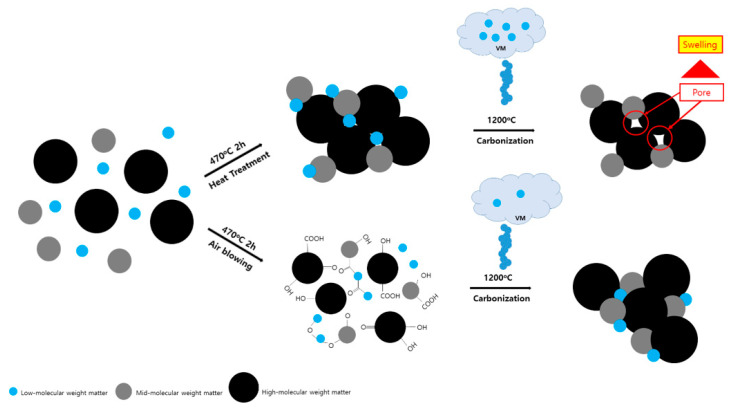
Schematic diagram.

Swelling occurred, and the microstructure was unstable in the cross-section when the sample was manufactured only in a nitrogen atmosphere. When considered in relation to the mechanical properties listed in Table 5, this phenomenon is judged to be due to the effect of oxygen during heat treatment. The postulated effect of air during the manufacture of binderless cokes is shown schematically in Figure 7. When only nitrogen is supplied during heat treatment to produce a binderless coke, a large amount of volatile matter remains, and when carbonized, the volatile matter rapidly escapes, and pores are formed inside the carbon block, which causes swelling. On the other hand, when air is allowed to flow, oxygen in the air causes a cross-linking reaction, reducing the amount of low molecular weight volatile matter and generating functional groups, such as hydroxyl, carbonyl, and carboxyl groups, which are advantageous for sintering. The carbon block will consequently have a more precise structure. In addition, an appropriate air ratio increases the β-resin content and suppresses the swelling phenomenon after carbonization, enabling the production of high-density, high-strength carbon blocks.

## 4. Conclusions

Through a study of the effect of the oxygen content of binderless coke produced from coal tar pitch on the mechanical properties of high-density carbon blocks, the following results were obtained. It was found that the volatile content decreased, and the fixed carbon content gradually increased as the air flow rate increased. The thermal stability was displayed in the order of N0A10 > N3A7 > N5A5 > N7A3 > N10A0, and the higher the air content ratio was, the higher the thermal stability was. As the air flow rate ratio increased, the C1s atomic% decreased, and O1s atomic% increased. In particular, the ratio of O1s/C1s indicated that the raw material was 6.19%, while the ratio of N0A10 was about twice as high at 11.20%. In particular, the atomic% of C–O (C1s) appeared more prominently than that of O–C=O. As the oxygen content increased, the structure of binderless coke was dense, and the mechanical strength increased after carbonization and swelling of the green body was suppressed.

## Figures and Tables

**Table 1:** Proximate analysis of raw material.

Sample Name	Proximate Analysis (wt%)			
	M ^a^	VM ^b^	Ash	FC ^c^
Special Pitch	0.04	18.28	0.19	81.49

^a^ M: Moisture, ^b^ VM: Volatile Matter, ^c^ FC: Fixed Carbon.

**Table 2:** Change in binderless coke yield according to flow ratio of nitrogen and air.

Sample Name	Raw Material	N_2_/Air (cc/min)	Temperature (°C)	Treating Time (h)	Yield (wt%)
N10A0	Special pitch	100/0	470	2	94.4
N7A3		70/30			91.3
N5A5		50/50			90.9
N3A7		30/70			91.6
N0A10		0/100			91.0

**Table 3:** Proximate analysis of binderless cokes manufactured according to flow ratio of nitrogen and air.

Proximate Analysis (wt%)				
Sample Name	M ^a^	VM ^b^	Ash	FC ^c^
N10A0 ^d^	1.30	8.61	0.65	89.44
N7A3	1.26	7.35	0.43	90.96
N5A5	1.13	7.34	0.40	91.13
N3A7	1.55	6.69	0.37	91.39
N0A10	1.57	6.58	0.39	91.46

^a^ M: Moisture, ^b^ VM: Volatile Matter, ^c^ FC: Fixed Carbon, ^d^ N_2_ 100cc/min/Air 0cc/min.

**Table 4:** Atomic% of C1s and O1s from X-ray photoelectric spectroscopy (XPS).

Atomic%								
Sample Name	C–C (C1s)	O–C=O (C1s)	C–O (C1s)	C1s	C–O (O1s)	O–C=O (O1s)	O1s	O1s/C1s
Raw pitch	67.6	2.16	24.41	94.17	5.59	0.24	5.83	6.19
N10A0	64.66	2.93	25.88	93.47	5.89	0.64	6.53	6.97
N7A3	58.93	2.87	31.39	93.19	5.29	1.52	6.81	7.31
N5A5	44.24	3.87	44.76	92.87	6.9	0.23	7.13	7.68
N3A7	42.62	4.1	42.12	90.84	7.24	1.92	9.16	10.08
N0A10	45.47	4.33	40.14	89.94	7.64	2.43	10.07	11.20

**Table 5:** Mechanical properties of high-density carbon according to the flow rate of air.

Sample Name	Molding Pressure (MPa)	Green Density (g/cm^3^)	Density (g/cm^3^)	Flexural Strength (MPa)	Shore Hardness (HSD)
CB-N10A0	150	1.324	1.518	44	62
CB-N7A3		1.335	1.675	60	84
CB-N5A5		1.338	1.700	77	89
CB-N3A7		1.346	1.718	79	92
CB-N0A10		1.365	1.724	87	93

## Data Availability

The data presented in this study are available on request from the corresponding author.
